# Epidemiology of lumbar punctures in hospitalized patients in the United States

**DOI:** 10.1371/journal.pone.0208622

**Published:** 2018-12-13

**Authors:** Adrienne Vickers, John P. Donnelly, Justin Xavier Moore, Scott R. Barnum, Theresa N. Schein, Henry E. Wang

**Affiliations:** 1 University of South Alabama School of Medicine, Mobile, Alabama, United States of America; 2 Department of Emergency Medicine, University of Alabama School of Medicine, Birmingham, Alabama, United States of America; 3 Department of Learning Health Sciences, University of Michigan Medical School, Ann Arbor, MI, United States of America; 4 Department of Surgery, Washington University School of Medicine, Saint Louis, MO, United States of America; 5 CNine Biosolutions, LLC., Birmingham, Alabama, United States of America; 6 Department of Emergency Medicine, University of Texas Health Science Center at Houston, Houston, Texas, United States of America; University of Pennsylvania Perelman School of Medicine, UNITED STATES

## Abstract

**Objectives:**

Lumbar puncture (LP) is an important technique for assessing and treating neurological symptoms. The objective of this study was to describe the characteristics of diagnostic lumbar punctures performed on hospitalized patients in the United States.

**Methods:**

We analyzed data from the 2010 National Inpatient Sample (NIS) and the National Emergency Department Survey (NEDS). We included patients treated in the Emergency Department (ED) as well as those admitted to an inpatient bed through the ED. We identified patients undergoing LPs from ICD-9 procedural code 03.31 and CPT code 62270. We generated nationally weighted estimates of the total number of LPs. We also assessed patient and hospital characteristics of cases undergoing LP.

**Results:**

Of an estimated 135 million hospitalizations (ED + admission, or ED only), there were an estimated 362,718 LPs (331,248–394,188), including 273,612 (251,850–295,375) among adults and 89,106 (71,870–106,342) among children (<18 years old). Of the 362,718 LPs, 136,764 (122,117–151,410) were performed in the ED without admission. The most common conditions associated with LP among children were fever of unknown origin, meningitis, seizures and other perinatal conditions. The most common conditions associated with LP among adults were headache and meningitis.

**Conclusions:**

Lumbar Puncture remains an important procedure for diagnostic and therapeutic uses in United States Hospitals.

## Introduction

The lumbar puncture was first introduced by Heinrich Quinke in 1891 to relieve elevated intracranial pressure in cases of meningitis.[1, 2] Since its innovation, the role of the LP has expanded to encompass diagnostic roles, including the identification of life-threatening conditions such as meningitis and hydrocephalus. The role of the LP is particularly important in the diagnosis of acute bacterial meningitis, which requires rapid diagnosis to prevent morbidity and mortality.[3, 4] Although more recent advances in imaging enhanced the diagnosis of neurologic emergencies, these modalities may miss conditions identifiable from cerebrospinal fluid analysis. For example, computed tomography imaging cannot capture microscopic bleeding from cerebral aneurysms. More importantly, radiologic imaging cannot prove the presence or absence of meningitis. Thus, the procurement of CSF from an LP remains an important tool for diagnosing many important neurologic emergencies.

However, the LP is an invasive procedure and has important pitfalls. A meta-analysis found that 1 in 3 patients undergoing the procedure may develop a post-LP headache.[5] Cerebral herniation occurs in 5% of patients with acute bacterial meningitis, with a strong temporal association with the performance of the LP even after a negative CT scan.[6, 7] Anticoagulant use (including novel oral anticoagulants) is a contraindication to LP, and the use of these agents in routine outpatient care is increasing.[8–10] Additionally, successfully performing a LP may be more difficult in obese patients and those with abnormal spinal anatomy.[11]

There have been no national descriptions of the epidemiology of LPs in United States (US) Emergency Departments (EDs); for example, the number of patients undergoing diagnostic LP, and their characteristics, diagnoses and outcomes. An awareness of these patterns is important for several reasons. The total number of cases would indicate equipment and training resources needed to adequately provide this procedure. These figures would indicate the number of patients vulnerable to LP adverse events, aiding in the planning for rescue therapies and opportunities for more advanced techniques such as ultrasound or fluoroscopically-guided LP. Novel biomarkers have been developed to help differentiate bacterial from viral CSF infections; epidemiologic descriptions of LPs could indicate the number of patients potentially benefitting from these novel assays.

In this study, we sought to describe the epidemiology of diagnostic LPs among Emergency Department-treated patients the United States.

## Methods

### Study design

We analyzed data from the Nationwide Emergency Department Sample (NEDS). This study was classified as not human subjects research by the Institutional Review Board of the University of Alabama at Birmingham.

### Survey content and administration

A component of the Healthcare Cost Utilization Project (HCUP), NEDS is the largest all-payer ED database in the US. NEDS combines national information from State Inpatient Databases (SIDs) and State Emergency Department Databases (SEDDs) and hence reflects patients treated in the ED and discharged or admitted to the hospital through an ED visit.[12] NEDS is an approximately 20% stratified sample of US hospital-based EDs entailing data from 945 hospitals in 33 states.[13] The NEDS data correspond to roughly 135 million ED visits annually. The NEDS database contains more than 100 clinical and nonclinical variables for each hospital visit or stay, including ED diagnoses, procedures, injury information, admission and discharge status, patient demographics, payer source, ED charges, and hospital characteristics.

NEDS encompasses ED-treated patients, including a) patients treated in the ED and subsequently admitted to the hospital, and b) patients treated in and subsequently discharged or transferred. We used NEDS data for the year 2010 because it provides the largest sample population and, therefore, is most appropriate for estimating total regional and national populations.

### Selection of patients

We identified all NEDS visits associated with the performance of a diagnostic LP. We identified LPs using ICD-9-CM procedure code 03.31 and Current Procedural Terminology code and Healthcare Common Procedure Coding System (CPT/HCPCS) procedure code 62270. We focused on diagnostic LPs because of our interest in patients treated in the ED for neurologic emergencies such as meningitis or subarachnoid hemorrhage. We excluded therapeutic LPs (CPT 62272) because these interventions are less common among ED-treated patients and are usually limited to patients with chronic neurologic conditions such as hydrocephalus.

It is important to note that the structure of the NEDS did not indicate the hospital location of procedures. Therefore, for patients initially treated in the ED and subsequently admitted, we could not differentiate if the LP was performed in the ED or in the inpatient setting. In contrast, LPs performed on patients who were treated only in ED could be more clearly attributed to the ED setting.

### Data analysis

Using descriptive statistical techniques, we calculated the number and incidence of LPs performed in the United States Emergency Departments in 2010. Following standard NEDS weighting procedures, we produced nationally weighted estimates of LPs and their corresponding 95% confidence intervals. For age-related estimates, we categorized patients as children (<18 years) or adults (≥18 years). In sensitivity analyses we further stratified the pediatric population to the age categories 0 to 5 years, 6 to 10 years, and 11 to 17 years.

We characterized all hospitalized patients by age, sex, and region of residence, along with household income quartile based on the residential zip code. We further differentiated LP patients by their primary diagnosis, obtained through classification based on Clinical Classifications Software (CCS) code at discharge. We characterized hospitals according to geographic region, teaching status, trauma center designation, and population setting (urban versus rural, defined by population estimates). In order to account for any seasonal variation in ED use, we also characterized ED visits by season. ED patient outcomes were categorized as routine discharge, inpatient admission, death, transferred to another hospital, or “other”, which includes transfers to nursing homes or other locations and left against medical advice. We also classified inpatient outcomes for patients admitted to the hospital of ED presentation using similar categories.

All analyses were performed using Stata v. 14.1 (Stata, Inc., College Station, TX).

## Results

During the study period, in the NEDS data set there were an estimated 135 million ED visits in the United States. There were a total of 362,718 LPs (incidence 2.7 per 1,000 ED visits), including 273,612 (75%) among adults and 89,106 (25%) among children. (Table 1) Among children, the majority were performed on patient ≤5 years old. Among the performed LPs, 136,764 (38%) were known to have occurred in the ED.

**Table 1:** National estimates of lumbar punctures performed for Emergency Department-treated patients, 2010.

	Emergency Department Admitted Inpatients^1^		Emergency Department Only Patients^2^	
Patient Group	No. of Raw Observations	Estimated Number of Lumbar Punctures (95% CI)	No. of Raw Observations	Estimated Number of Lumbar Punctures (95% CI)
**All Patients**	79,697	362,718 (331,248–394,188)	30,045	136,764 (122,117–151,410)
**Adult Patients (Age ≥18 years)**	60,016	273,612 (251,850–295,375)	22,913	105,015 (93,268–116,762)
**Children**				
0–5 years	14,360	64,849 (50,703–78,995)	4,104	18,096 (15,047–21,144)
6–10 years	1,707	7,762 (6,321–9,203)	833	3,736 (3,181–4,294)
11–17 years	3,614	16,495 (14,357–18,633)	2,195	9,915 (8,723–11,108)

^1^Includes patients who were treated in the ED and subsequently admitted to the hospital.

^2^Includes only patients who treated in and subsequently discharged from the ED

Among children, patients undergoing LP were more likely to be male than female. (Table 2) In contrast, gender distributions were similar for adults. For both age groups, LPs were more common for urban than rural patients. The incidence of lumbar puncture was also higher among patients from lower income areas. Among adults, LPs were most common in hospitals in the south census region. Though this result was not seen for all children, it was appreciated in our sensitivity analysis for younger children ≤5 years old. (S1 Appendix) Among all children, LPs were more common in trauma centers. Regardless of age, LPs were more common in urban and teaching hospitals and more common during summer months.

**Table 2:** Patient and hospital characteristics of Emergency Department-treated patients by age and lumbar puncture status, 2010.

	Patients <18 Years Old			Patients ≥18 Years Old		
Characteristic	Lumbar Puncture (N = 89,106)	No Lumbar Puncture (N = 25,421,964)	P-value	Lumbar Puncture (N = 273,612)	No Lumbar Puncture (N = 103,176,378)	P-value
**Patient Sex**			<0.001			0.21
Male	55.0 (54.3–55.7)	52.7 (52.6–52.9)		42.2(41.6–42.9)	42.6 (42.3–42.9)	
Female	45.0 (44.3–45.7)	47.3 (47.1–47.4)		57.8(57.1–58.4)	57.4 (57.1–57.7)	
**Patient Residence**			<0.001			<0.001
Urban	88.6 (86.4–90.4)	79.7 (78.0–81.2)		87.1(85.5–88.6)	80.1 (78.8–81.3)	
Rural	11.4 (9.6–13.6)	20.3 (18.8–22.0)		12.9 (11.4–14.5)	19.9 (18.7–21.2)	
**Patient Median Income**			0.004			<0.001
$1–40,999	28.5 (23.9–33.6)	32.9 (30.1–35.8)		26.6 (24.1–29.1)	33.2 (30.9–35.6)	
$41,000-$50,999	27.7 (25.3–30.3)	29.0 (26.9–31.2)		27.1 (25.3–29.0)	27.8 (26.1–29.4)	
$51,000-$66,999	24.8 (21.7–28.2)	22.0 (20.23–23.9)		25.0 (22.6–27.6)	21.7 (20.3–23.3)	
$67,000 or more	19.0 (16.5–21.9)	16.0 (14.2–18.1)		21.3 (19.0–23.7)	17.3 (15.6–19.1)	
**Hospital Census Region**			<0.001			<0.001
Northeast	13.4 (9.6–18.4)	18.1 (15.8–20.7)		16.2 (13.4–19.6)	19.5 (17.9–21.1)	
Midwest	15.7 (11.1–21.8)	22.4 (19.9–25.2)		21.5 (18.7–24.6)	23.8 (21.9–25.8)	
South	40.1 (30.8–50.2)	39.6 (36.3–43.1)		37.3 (33.6–41.2)	39.4 (37.4–41.4)	
West	30.8 (22.2–41.1)	19.9 (17.3–22.8)		25.0 (21.5–28.7)	17.4 (16.0–18.8)	
**Hospital Population Setting**			<0.001			<0.001
Urban	93.2 (91.3–94.8)	80.7 (79.0–82.3)		91.6 (90.1–92.9)	81.8 (80.5–83.0)	
Rural	6.8 (5.3–8.7)	19.3 (17.8–21.1)		8.4 (7.2–9.9)	18.2 (17.0–19.5)	
**Hospital Teaching Status**			<0.001			<0.001
Metropolitan non-teaching	28.1 (22.4–34.6)	40.6 (37.6–43.7)		38.7(35.3–42.3)	42.4 (40.4–44.4)	
Metropolitan teaching	65.1 (57.7–71.9)	40.0 (36.3–43.9)		52.9 (49.0–56.7)	39.4 (37.2–41.6)	
Non-metropolitan teaching and non-teaching	6.8 (5.3–8.7)	19.3 (17.8–21.1)		8.4 (7.2–9.9)	18.2 (17.0–19.5)	
**Hospital Trauma Center**			<0.001			<0.001
Trauma center	59.1 (49.5–68.0)	38.4 (34.9–42.1)		48.5 (44.5–52.5)	35.7 (33.6–37.4)	
Not trauma center	41.0 (32.1–50.5)	61.6 (57.9–65.1)		51.5 (47.5–55.5)	64.4 (62.2–66.4)	
**Admission by Season**			<0.001			0.001
Spring	23.8 (23.0–24.7)	26.2 (26.1–26.3)		24.8 (24.3–25.3)	25.3 (25.2–25.5)	
Summer	27.9 (26.7–29.0)	23.9 (23.8–24.1)		26.8 (26.3–27.3)	26.3 (26.2–26.4)	
Fall	25.1 (24.3–26.0)	24.9 (24.7–25.1)		25.4 (24.8–26.0)	24.7 (24.6–24.8)	
Winter	23.2 (22.3–24.1)	24.9 (24.8–25.1)		23.0 (22.6–23.5)	23.7 (23.6–23.8)	

Table cells reflect column totals and 95% confidence intervals.

Among children, fever of unknown origin, other perinatal conditions, meningitis, and seizures were the more common conditions associated with LP. (Table 3) Among adults, headache and meningitis accounted for more than half of the hospitalizations associated with an LP. Most children receiving LPs were discharged after being admitted, while a large majority of children not receiving LPs were discharged home from the ED. (Table 4) However, similar proportions of adults were routinely discharged directly from the ED and routinely discharged after admission. Among patients receiving LPs, a larger proportion of adults died following admission as compared with children, but causes of death are unknown. Results of outcomes comparisons were similar when stratifying by age for pediatric patients. (S2 Appendix)

**Table 3:** Primary diagnosis groupings among Emergency Department-treated patients undergoing lumbar puncture by age, 2010.

Diagnoses (CCS Codes)	Patients <18 Years (N = 89,106)	Patients ≥18 Years (N = 273,612)
Headache; including migraine 84	8.6 (7.7–9.7)	39.9 (38.2–41.6)
Meningitis (excluding tuberculosis or sexually transmitted diseases) 76	13.6 (12.4–14.8)	12.7 (12.2–13.3)
Fever of unknown origin 246	20.4 (19.1–21.8)	4.7 (4.3–5.0)
Epilepsy; convulsions 83	11.2 (10.3–12.1)	5.4 (5.1–5.8)
Septicemia 2	5.3 (4.7–5.9)	7.5 (7.0–8.1)
Other nervous system disorders 95	2.1 (1.8–2.5)	8.8 (8.2–9.5)
Other perinatal conditions 224	17.9 (15.8–20.3)	-
Viral Infection 7	7.0 (6.2–7.8)	3.4 (3.2–3.6)
Urinary tract infections 159	5.8 (5.2–6.5)	2.1 (2.0–2.3)
Acute cerebrovascular disease 109	0.04 (0.03–0.05)	3.8 (3.5–4.2)
Other upper respiratory infections 126	4.0 (3.6–4.5)	2.1 (1.9–2.3)
Pneumonia (excluding tuberculosis or sexually transmitted diseases) 122	2.5 (2.2–2.8)	2.4 (2.2–2.6)
Encephalitis (excluding tuberculosis or sexually transmitted diseases) 77	0.8 (0.7–1.0)	2.4 (2.2–2.6)
Spondylosis; intervertebral disc disorders; other back problems 205	0.6 (0.5–0.8)	1.9 (1.7–2.0)

Table cells reflect column totals and 95% confidence intervals.

CCS = Clinical Classification Software

**Table 4:** Disposition of Emergency Department-treated patients by age and lumbar puncture status, 2010.

	Patients <18 Years Old		Patients ≥18 Years Old	
Emergency Department Disposition	Lumbar Puncture (N = 89,106)	No Lumbar Puncture (N = 25,421,964)	Lumbar Puncture (N = 273,612)	No Lumbar Puncture (N = 103,176,378)
Routine discharge	25.6 (23.0–28.3)	93.6 (93.1–94.0)	36.0 (33.8–38.2)	77.3 (76.7–77.9)
Transfer to short term hospital	8.9 (6.9–11.3)	1.2 (1.1–1.3)	1.5 (1.3–1.8)	1.6 (1.4–1.7)
Died in ED	N/A	0.03 (0.03–0.04)	N/A	0.2 (0.2–0.2)
Other	N/A	1.4 (1.1–1.8)	N/A	3.2 (2.9–3.5)
Admit				
Routine discharge	59.2 (54.7–63.5)	3.5 (3.1–3.9)	36.9 (35.6–38.2)	11.2 (10.8–11.5)
Transfer to short term hospital	1.9 (1.5–2.3)	0.08 (0.08–0.09)	2.5 (2.3–2.8)	0.5 (0.5–0.5)
Died on ward	0.2 (0.2–0.3)	0.01 (0.01–0.02)	2.1 (1.9–2.3)	0.5 (0.5–0.5)
Other	3.3 (2.4–4.4)	0.2 (0.15–0.26)	22.2 (20.9–23.6)	6.3 (6.1–6.5)

Table cells denote column percentages and 95% confidence intervals. N/A = not available due to n<11.

## Discussion

Our results indicate that out of an estimated 135 million Emergency Department hospitalizations reported in the 2010 NEDS data, approximately 362,718 were associated with a diagnostic LP. These figures suggest that approximately 3 of every 1,000 ED treatments may be associated with a diagnostic LP in the ED or subsequent inpatient course. Furthermore, acknowledging the limitations of the data set, at least 37% of the total estimated LPs were performed in the ED. Though the majority of LPs were performed on adults, an estimated 64,849 (18% of total) were performed on children ≤5 years old. These observations characterize the frequency and burden of LPs in contemporary hospital practice.

There have been few prior descriptions of LP epidemiology among either ED or hospitalized patients. Shah, et al. examined the incidence of traumatic lumbar punctures in the ED, but only included patients from a single tertiary hospital ED.[14] Kroll, et al. evaluated US LP trends by medical specialty over span of 20 years but did not include detailed patient or institutional information from the ED.[15] Our study provides one of the first national descriptions of LP performance among patients receiving initial treatment in the ED.

An interesting observation was the disproportionately higher number of younger pediatric and adult LPs in the Southern US census region, which may be due to regional variations in disease presentation or practice. For example, the incidence of suspected meningitis may be higher in the South census region. Wang, et al. observed that sepsis mortality was highest in the Southeast US, potentially explaining the higher frequency of LPs.[16] We also observed a higher proportion of LPs among low income patients. We do not know whether these disparities are due to differences in disease prevalence or clinical practices. More targeted epidemiological studies of these specific factors could be warranted to evaluate whether this is a concern of public health or clinical practice.

In highlighting the national number of procedures, our epidemiologic observations illuminate opportunities for improving the care of patients requiring LPs. LP is a complex, invasive procedure with potentially serious complications and challenges in the face of difficult patient anatomy such as obesity or distorted spinal anatomy. Novel LP techniques include the use of adjunctive fluoroscopy, CT, and ultrasonography to facilitate LP performance. Our observations suggest that a considerable number of patients may benefit from these approaches. Novel biomarkers may identify the early stages of cerebral infection and neurodegenerative diseases.[17–19] For example, some complement activation components and the soluble membrane attack complex show promise as biomarkers of bacterial meningitis and shunt infection in patients with hydrocephalus.[20, 21] Given that LPs are most commonly used to rule out bacterial meningitis, the potential for the application of these diagnostic biomarkers is large.

## Limitations

This study has several limitations. NEDS aggregates patients treated only in the ED as well as those admitted through the ED. Diagnostic LPs performed on an outpatient basis were not included in this study. Among admitted patients, we could not differentiate LPs occurring in the ED vs. those performed later in the hospital course. Thus, our observed number of ED LP’s may be an underestimate. As a result of this, we also could not assess the specialty of the provider who performed the LP nor whether the admission was a result of the LP or not. We could not ascertain the use or success of different LPs approaches such as patient positioning or the use of varying LP needles.

We were limited to 2010 data and could not evaluate secular trends. Although the NEDS contain comprehensive data, variations in documentation practices may have led to underestimates of the number of LPs. We did not have data on adverse events (such as post-dural headaches) and, therefore, also could not distinguish whether the discharge diagnosis, such as a headache, was the indication for the LP, or a result of the LP. Finally, although we had CCS for each case, these codes do not indicate the reason for an LP. Diagnoses such as pneumonias or UTIs may have denoted instances of sepsis workups where a diagnostic LP was performed as part of the work-up. Alternatively, the LP may have been done later in response to additional developing symptoms.

## Conclusions

Approximately 363,000 LPs are performed each year among ED-treated patients in the United States. These observations highlight the national number of diagnostic LP interventions and the opportunities for improving the care of these patients with potential neurologic emergencies.

## Supporting information

S1 Appendix Patient and hospital characteristics of Emergency Department-treated patients undergoing lumbar puncture, stratified by age. (DOCX)

S2 Appendix Disposition of Emergency Department-treated patients by age and lumbar puncture status with additional stratification, 2010. (DOCX)
