# Supplementary material for: Epidemiology of lumbar punctures in hospitalized patients in the United States
Source: PLoS One. 2018 Dec 13;13(12):e0208622. doi: 10.1371/journal.pone.0208622 (PMC6292631; doi:10.1371/journal.pone.0208622)
Supplement: S1 Appendix — (DOCX) [file pone.0208622.s001.docx]

# S1 Appendix 1

Patient and hospital characteristics of Emergency Department-treated patients undergoing lumbar puncture, stratified by age.

|  | **Age Group** | | | | | | | |
| --- | --- | --- | --- | --- | --- | --- | --- | --- |
|  | **0 – 5 Years** | | **6 – 11 Years** | | **12 – 17 Years** | | **≥18 Years** | |
| **Characteristic** | **LP (%)**  **(N = 64,849)** | **No LP (%)**  **(N =**  **12,634,367)** | **LP (%)**  **(N = 7,762)** | **No LP (%)**  **(N =**  **4,827,334)** | **LP (%)**  **(N = 16,495)** | **No LP (%)**  **(N =**  **7,960,263)** | **LP (%)**  **(N = 273,612)** | **No LP (%)**  **(N =**  **103,176,378)** |
| **Patient Sex (%)** | |  |  |  |  |  |  |  |
| Male | 56.3  (55.5-57.1) | 54.5  (54.4-54.6) | 57.5  (55.0-59.9) | 53.8  (53.7-54.0) | 48.9  (46.8-50.9) | 49.3  (49.1-49.6) | 42.2  (41.6-42.9) | 42.6  (42.3-42.9) |
| Female | 43.7  (42.9-44.6) | 45.5  (45.4-45.6) | 42.5  (40.1-45.0) | 46.2  (46.0-46.3) | 51.1  (49.1-53.2) | 50.7  (50.4-50.9) | 57.8  (57.1-58.4) | 57.4  (57.1-57.7) |
| **Patient residence (%)** | | |  |  |  |  |  |  |
| Urban | 89.0  (86.8-90.9) | 80.4  (78.6-82.1) | 88.1  (85.1-90.6) | 79.4  (77.8-81.0) | 86.8  (84.2-89.1) | 78.6  (77.1-79.9) | 87.1 (85.5-88.6) | 80.1  (78.8-81.3) |
| Rural | 11.0 (9.1-13.2) | 19.6  (17.9-21.4) | 11.9  (9.4-14.9) | 20.6  (19.0-22.2) | 13.2  (10.9-15.8) | 21.5  (20.1-22.9) | 12.9 (11.4-14.5) | 19.9  (18.7-21.2) |
| **Median Income (%)** | |  |  |  |  |  |  |  |
| $1-40,999 | 30.7  (25.5-36.3) | 34.2  (31.2-37.4) | 25.9  (21.6-30.8) | 32.4  (29.7-35.3) | 21.2  (17.7-25.1) | 31.1  (28.6-33.7) | 26.6  (24.1-29.1) | 33.2  (30.9-35.6) |
| $41,000-$50,999 | 27.5  (24.9-30.4) | 30.0  (27.6-32.4) | 27.9  (24.5-31.6) | 28.7  (26.6-31.0) | 28.3  (25.8-31.0) | 27.7  (26.0-29.6) | 27.1  (25.3-29.0) | 27.8  (26.2-29.4) |
| $51,000-$66,999 | 24.2  (20.9-27.8) | 21.9  (20.0-24.0) | 25.6  (21.7-29.8) | 22.0  (20.2-23.9) | 26.8  (23.6-30.1) | 22.2  (20.6-24.0) | 25.0  (22.6-27.6) | 21.7  (20.3-23.3) |
| $67,000 or more | 17.6  (15.0-20.6) | 13.9  (12.2-15.8) | 20.6  (17.1-24.7) | 16.9  (14.9-19.0) | 23.8  (21.1-26.8) | 18.9  (16.9-21.2) | 21.3  (19.0-23.7) | 17.3  (15.6-19.1) |
| **Hospital Census Region (%)** | |  |  |  |  |  |  |  |
| Northeast | 13.9  (9.5-19.9) | 16.7  (14.0-19.8) | 10.6  (7.6-14.6) | 18.7  (16.3-21.3) | 12.6  (10.1-15.5) | 20.0  (18.1-22.1) | 16.2  (13.4-19.6) | 19.5  (17.9-21.1) |
| Midwest | 14.3  (9.6-20.8) | 21.6  (18.7-24.8) | 18.5  (13.1-25.6) | 22.6  (20.0-25.4) | 20.1  (15.8-25.2) | 23.5  (21.4-25.8) | 21.5  (18.7-24.6) | 23.8  (21.9-25.8) |
| South | 42.9  (32.4-54.1) | 40.4  (36.4-44.5) | 34.8  (26.6-44.1) | 40.2  (36.8-43.6) | 31.6  (25.4-38.4) | 38.0  (35.4-40.7) | 37.3  (33.6-41.2) | 39.4  (37.4-41.4) |
| West | 28.9  (19.4-40.7) | 21.3  (18.0-24.9) | 36.1  (27.1-46.2) | 18.6  (16.0-21.4) | 35.8  (29.7-42.4) | 18.4  (16.6-20.4) | 25.0  (21.5-28.7) | 17.4  (16.0-18.8) |
| **Hospital Population Setting (%)** | | |  |  |  |  |  |  |
| Urban | 93.8  (91.4-95.3) | 81.6  (79.8-83.4) | 93.2  (90.8-95.1) | 80.3  (78.5-81.9) | 91.1  (88.5-93.1) | 79.4  (77.9-80.8) | 91.6  (90.1-92.9) | 81.8  (80.5-83.0) |
| Rural | 6.2  (4.7-8.2) | 18.4 (16.6-20.3) | 6.8  (5.0-9.2) | 19.7  (18.1-21.5) | 8.9  (6.9-11.5) | 20.6  (19.2-22.2) | 8.4  (7.2-9.9) | 18.4  (17.0-19.5) |
| **Hospital Teaching Status (%)** | | |  |  |  |  |  |  |
| Metropolitan non-teaching | 25.4  (19.6-32.2) | 39.0  (35.5-42.5) | 31.2  (24.8-38.4) | 41.3  (38.3-44.4) | 37.5  (32.2-43.1) | 42.9  (40.4-45.4) | 38.7  (35.3-42.3) | 42.4  (40.4-44.4) |
| Metropolitan teaching | 68.4  (60.6-75.3) | 42.7  (38.3-47.2) | 62.1  (54.2-69.4) | 39.0  (35.2-42.9) | 53.6  (47.4-59.7) | 36.5  (33.6-39.5) | 52.9  (49.0-56.7) | 39.4  (37.2-41.6) |
| Non-metropolitan teaching and non-teaching | 6.2  (4.7-8.2) | 18.4  (16.6-20.3) | 6.8  (5.0-9.2) | 19.7  (21.5-18.1) | 8.9  (6.9-11.5) | 20.0  (19.2-22.2) | 8.4  (7.2-9.9) | 18.2  (17.0-19.5) |
| **Hospital Trauma Level (%)** | |  |  |  |  |  |  |  |
| Non-trauma | 39.2  (29.4-50.0) | 59.3  (55.0-63.5) | 43.1  (34.3-52.4) | 62.6  (58.9-66.2) | 46.8  (40.5-53.2) | 64.6  (61.8-67.2) | 51.5  (47.5-55.5) | 64.4  (62.2-66.4) |
| Trauma | 60.8  (50.0-70.6) | 40.7  (36.5-45.0) | 56.9  (47.6-65.7) | 37.4  (33.9-41.1) | 53.2  (46.8-59.5) | 35.5  (32.8-38.2) | 48.5  (44.5-52.2) | 35.7  (33.6-37.8) |
| **Admissions (%)** | |  |  |  |  |  |  |  |
| Spring | 24.4  (23.4-25.4) | 25.7  (25.5-25.8) | 21.9  (19.8-24.1) | 27.2  (27.0-27.4) | 22.7  (21.2-24.4) | 26.4  (26.2-26.5) | 24.8  (24.3-25.3) | 25.3  (25.2-25.5) |
| Summer | 26.4  (25.2-27.6) | 23.3  (23.1-23.5) | 35.7  (32.6-38.9) | 25.2  (24.9-25.4) | 29.8  (27.8-31.8) | 24.2  (24.0-24.4) | 26.8  (26.3-27.3) | 26.3  (26.2-26.4) |
| Fall | 24.5  (23.5-25.5) | 23.8  (23.6-24.0) | 25.5  (23.2-28.0) | 24.7  (24.4-24.9) | 27.4  (25.2-29.6) | 26.8  (26.6-27.0) | 25.4  (24.8-26.0) | 24.7  (24.6-24.8) |
| Winter | 24.8  (23.7-25.9) | 27.2  (27.0-27.4) | 16.9  (14.7-19.4) | 23.0  (22.8-23.2) | 20.1  (18.7-21.7) | 22.6  (22.4-22.7) | 23.0  (22.6-23.5) | 23.7  (23.6-23.8) |
|  |  |  |  |  |  |  |  |  |

Table cells reflect column percentages and 95% confidence intervals.
