# Supplementary material for: Epidemiology of lumbar punctures in hospitalized patients in the United States
Source: PLoS One. 2018 Dec 13;13(12):e0208622. doi: 10.1371/journal.pone.0208622 (PMC6292631; doi:10.1371/journal.pone.0208622)
Supplement: S2 Appendix — (DOCX) [file pone.0208622.s002.docx]

# S2 Appendix

Disposition of Emergency Department-treated patients by age and lumbar puncture status with additional stratification, 2010.

|  | **Age Group** | | | | | | | |
| --- | --- | --- | --- | --- | --- | --- | --- | --- |
|  | **0 – 5 Years** | | **6 – 10 Years** | | **11 – 17 Years** | | **≥18 Years** | |
| **Disposition** | **LP (%)**  **(N = 64,849)** | **No LP (%)**  **(N =**  **12,634,367)** | **LP (%)**  **(N = 7,762)** | **No LP (%)**  **(N =**  **4,827,334)** | **LP (%)**  **(N = 16,495)** | **No LP (%)**  **(N =**  **7,960,263)** | **LP (%)**  **(N = 273,612)** | **No LP (%)**  **(N =**  **103,176,378)** |
| **Disposition of Emergency Department Patients** | |  |  |  |  |  |  |  |
| Routine discharge | 17.3  (15.2-19.6) | 93.7  (93.2-94.2) | 38.9  (34.3-43.8) | 94.8  (94.3-95.2) | 51.7  (48.2-55.3) | 92.6  (92.1-93.1) | 36.9  (33.8-38.2) | 77.3  (76.7-77.9) |
| Transfer to short term hospital | 9.4  (7.1-12.3) | 1.2  (1.1-1.3) | 8.0  (6.1-10.4) | 1.1  (1.0-1.2) | 7.3  (5.8-9.3) | 1.4  (1.3-1.5) | 1.5  (1.3-1.8) | 1.6  (1.4-1.7) |
| Died in ED | N/A | 0.04  (0.04-0.05) | N/A | 0.01  (0.01-0.02) | N/A | 0.03  (0.02-0.03) | N/A | 0.19  (0.18-0.19) |
| Other | N/A | 1.1  (1.0-1.6) | 1.2  (0.7-2.2) | 1.2  (0.9-1.5) | 1.1  (0.7-1.6) | 1.9  (1.6-2.3) | N/A | 3.2  (2.9-3.5) |
| **Disposition of Admitted Patients** | |  |  |  |  |  |  |  |
| Routine discharge | 67.1  (62.9-71.0) | 3.6  (3.2-4.1) | 45.9  (40.5-51.3) | 2.8  (2.4-3.2) | 34.4  (30.5-38.5) | 3.7  (3.3-4.1) | 36.8  (35.6-38.2) | 11.2  (10.8-11.5) |
| Transfer to short term hospital | 1.9  (1.5-2.5) | 0.1  (0.1-0.1) | 2.0  (1.4-2.8) | 0.1  (0.1-0.1) | 1.8  (1.3-2.3) | 0.1  (0.1-0.1) | 2.5  (2.3-2.8) | 0.5(0.5-0.5) |
| Died on ward | 0.2  (0.2-0.3) | 0.01  (0.01-0.02) | N/A | 0.01  (0.01-0.01) | N/A | 0.02  (0.01-0.02) | 2.1  (1.9-2.3) | 0.5  (0.5-0.5) |
| Other | 3.1  (2.3-4.2) | 0.2  (0.1-0.2) | 4.0  (2.5-6.3) | 0.1  (0.1-0.2) | 3.8  (2.8-5.1) | 0.3  (0.2-0.3) | 22.2  (20.9-23.6) | 6.3  (6.1-6.5) |
|  | | | | | | | | |

Table cells reflect column percentages and 95% confidence intervals. N/A = not available due to n<11.
